# Shifting GnRH Neuron Ensembles Underlie Successive Preovulatory Luteinizing Hormone Surges

**DOI:** 10.1523/JNEUROSCI.1383-24.2024

**Published:** 2024-11-06

**Authors:** Shel-Hwa Yeo, Su Young Han, Allan E. Herbison

**Affiliations:** Department of Physiology, Development and Neuroscience, University of Cambridge, Cambridge CB2 3EG, United Kingdom

**Keywords:** ensemble, fertility, GnRH, LH surge

## Abstract

The gonadotropin-releasing hormone (GnRH) neurons operate as a neuronal ensemble exhibiting coordinated activity once every reproductive cycle to generate the preovulatory GnRH surge. Using GCaMP fiber photometry at the GnRH neuron distal dendrons to measure the output of this widely scattered population in female mice, we find that the onset, amplitude, and profile of GnRH neuron surge activity exhibits substantial variability from cycle to cycle both between and within individual mice. This was also evident when measuring successive proestrous luteinizing hormone surges. Studies combining short (c-Fos and c-Jun) and long (genetic robust activity marking) term indices of immediate early gene activation revealed that, while ∼50% of GnRH neurons were activated at the time of each surge, only half of these neurons had been active during the previous proestrous surge. These observations reveal marked inter- and intra-individual variability in the GnRH surge mechanism. Remarkably, different subpopulations of overlapping GnRH neurons are recruited to the ensemble each estrous cycle to generate the GnRH surge. While engendering variability in the surge mechanism itself, this likely provides substantial robustness to a key event underlying mammalian reproduction.

## Significance Statement

The midcycle luteinizing hormone (LH) surge driven by the gonadotropin-releasing hormone (GnRH) neurons represents the key event triggering ovulation in all mammals. Using GCaMP fiber photometry and genetic activation markers, we unexpectedly find that different subpopulations of GnRH neurons are responsible for driving consecutive LH surges every 4–5 d in cycling female mice. This remarkable oscillatory pattern of network plasticity within the ensemble occurs under normal physiological conditions and likely contributes to the variable timing of the onset of LH surge both within and between individuals. The ability of individual GnRH neurons to take turns within the ensemble in driving the LH surge likely provides a robust fail-safe mechanism for ovulation and contributes to the robustness of mammalian fertility.

## Introduction

It has become increasingly appreciated that ensembles of coordinated neurons represent key functional modules within the brain that underlie meaningful behavior (Yuste, 2015). One such ensemble is responsible for initiating ovulation in mammals and has the gonadotropin-releasing hormone (GnRH) neuronal phenotype as its final output neuron. Presumably as a result of their extraordinary developmental origin in the nose and subsequent migration into the brain (Duittoz et al., 2022), the GnRH neurons have many unusual properties including a dispersed topography in which their cell bodies are scattered throughout the basal forebrain (Herbison, 2015). By coupling with hypothalamic neurons modulated by gonadal steroids and providing circadian inputs, a subpopulation of these GnRH neurons becomes synchronously active at the midpoint of each reproductive cycle to generate the “GnRH surge” that triggers ovulation (Goodman et al., 2022).

The scattered distribution of the GnRH neurons has been a major obstacle to understanding the behavior of this functional ensemble in vivo. Nevertheless, GnRH portal bleeding and immediate early gene (IEG) histochemical studies have clearly demonstrated that ∼50% of all GnRH neurons are activated at the same time to generate a GnRH surge that lasts for over 12 h (Lee et al., 1990; Hoffman et al., 1993; Karsch et al., 1997). Beyond this, however, many fundamental questions remain regarding the precise identity, mechanisms of activation, and nature of coordinated activity among GnRH neurons (Goodman et al., 2022; Kauffman, 2022; Piet, 2023).

One curious feature of the proestrous luteinizing hormone (LH) surge is that the onset is highly variable between mice (Miller et al., 2004; Minabe et al., 2011; Czieselsky et al., 2016) despite the use of a precise circadian timing mechanism (Tonsfeldt et al., 2022; Piet, 2023). This may result from technical issues related to the variable impact of stress on the proestrous surge in experimental situations (Wagenmaker and Moenter, 2017) or, perhaps, reflect endogenous “jitter” in the activation of the GnRH neuron ensemble.

We recently developed a GCaMP fiber photometry approach that allows the activity of the dispersed GnRH neuron ensemble to be recorded at high resolution for several days in undisturbed freely behaving mice (Han et al., 2024). Using this approach, we first asked whether GnRH neuron activity at the time of the proestrous surge was indeed different between individual mice and found marked variability not only between mice but across successive surges in the same mice. Next, using a combination of conventional short-term and genetic longer-term markers of IEG activation, we identified that, while on average, 50% of GnRH neurons are activated at each surge, overlapping GnRH neuron ensembles are responsible for sequential surges in the same mice. This unexpected and highly unusual situation indicates that, under normal physiological conditions, different GnRH neurons are recruited to the ensemble generating each proestrous GnRH surge.

## Materials and Methods

### Animals

Female 129S6Sv/Ev C57BL/6 *Gnrh1^Cre/+^*mice (Yoon et al., 2005) alone or crossed on to the Ai162 (TIT2L-GC6s-ICL-tTA2)-D Cre-dependent GCaMP6s line (JAX stock #031562; Daigle et al., 2018) were group-housed in conventional cages with environmental enrichment under conditions of controlled temperature (22 ± 2°C) and lighting (12 h light/dark cycle; lights on at 07:00) with *ad libitum* access to food (RM1-P, Special Diets Services) and water. All animal experimental protocols were approved by the University of Cambridge Animal Welfare and Ethics Review Body (UK Home Office license, P174441DE).

### Stereotaxic implantation of optic fibers

Adult *Gnrh1^Cre/+^* GCaMP6 mice were anesthetized with 2% isoflurane and placed in a stereotaxic frame with buprenorphine (0.05 mg/kg, s.c.) and meloxicam (5 mg/kg, s.c.) analgesia. Dexamethasone (10 mg/kg, s.c.) was used to prevent cranial swelling. A single 400 μm diameter optic fiber (0.48 NA, Doric Lenses) was implanted into the brain with the tip placed immediately above the arcuate nucleus (AP to bregma, −2.0 mm; DV −5.9 mm; ML ±0.2 mm to sagittal sinus). Following one week of surgery, all animals were handled daily and habituated to a photometry recording setup for at least 3 weeks.

### GCaMP6 fiber photometry over successive proestrous days

Beginning from 4 weeks postsurgery, fiber photometry experiments were undertaken to record the GCaMP fluorescence signal in freely behaving mice over 24 h periods beginning on the morning of proestrus. Recordings were made between two to three successive proestrous stages. Vaginal smears were collected by flushing 4 µl of sterile phosphate-buffered saline into the vaginal orifice and then transferring the collected fluid to a glass slide before staining with filtered Giemsa. All smears were examined with a light microscope, and the smear stages were determined by the proportion of leukocytes, nucleated, or cornified cells.

The fiber photometry recordings were performed using previously described methodology (Han et al., 2019, 2023). This included a custom-built photometry system using Doric components (Doric Lenses) and a National Instruments data acquisition board based on a previous design (Lerner et al., 2015). Blue (465–490 nm) and violet (405 nm) LED lights were sinusoidally modulated at frequencies of 531 and 211 Hz, respectively, and were focused onto a single fiber optic connected to the mouse. The light intensity at the tip of the fiber was 30–80 μW. Emitted fluorescence from the brain was collected via the same fiber, passed through a 500–550 nm emission filter, and focused onto a fluorescence detector (Doric Lenses). The emissions were collected at 10 Hz, and the two GCaMP6 emissions were recovered by demodulating the 465–490 nm signals (calcium-dependent) and 405 nm (calcium-independent) signals. Signals were either recorded in a continuous mode or a scheduled 5 s on/10 s off mode. The calcium-dependent signal was isolated by subtracting the 405 nm signal from the 465–490 nm signal. An exponential fit algorithm was then applied to correct for baseline shifts due to any photobleaching. The resulting signal was converted to Δ*F*/*F* (%) values using the equation Δ*F*/*F* = [(F_recorded − F_baseline)/F_baseline] × 100, where F_recorded represents the recorded fluorescent signal and F_baseline is the exponential-fitted baseline at the corresponding time.

For the deconvolution of signals from 24 h proestrous recordings, a moving average (movmean) algorithm was utilized to calculate a 30 min rolling average. This approach was used to detect any slow oscillatory shifts in baseline activity during the surge. The onset, offset, and peak amplitude of this oscillatory activity were determined from the deconvoluted signals. The surge onset time was identified as the point at which a >5% increase in Δ*F* occurred relative to all previous time points. The offset of the surge signal was determined as being the time at which the calcium signal returned to 90% of the baseline value. The peak amplitude was defined as the highest value observed during the slow incremental phase of the surge.

### Adeno-associated virus (AAV)–robust activity marking injections

*Gnrh1^Cre/+^* female mice were anesthetized with isoflurane (4% induction, 2.0% maintenance during surgery) and received meloxicam (5 mg/kg, s.c.) and buprenorphine (0.05 mg/kg, s.c.) prior to placement in a Kopf Instruments stereotaxic apparatus. A custom-made bilateral Hamilton syringe apparatus holding two 25-gauge needles 0.9 mm apart was used to perform bilateral injections into the rostral preoptic area (rPOA) with the following coordinates: AP, +0.75; DV, −4.60. The needles were lowered into place over 2 min and left in situ for 3 min before the injection was made. Bilateral injections of 1.0 μm of AAV1-RAM-d2tTA-pA:TRE-FLEX-TdTomato-WPRE-pA (2.0 × 10^13^ GC/ml, VectorBuilder) were given into the rPOA at a rate of 60 nl/min, with the needles left in situ for 15 min before being withdrawn. Oral meloxicam (5 mg/kg body weight) was given to animals for postoperative pain relief for the first 2 d. Mice were allowed to recover for 5 d before commencing the doxycycline diet regime, and beginning 1 week after surgery, mice were handled daily and habituated for tail-tip bleeding.

### Doxycycline treatments, estrous cycle monitoring, and LH surge assessment

Mice receiving AAV1-RAM were kept on a doxycycline diet (40 mg doxycycline/kg; Envigo) for 10–14 d after recovering from surgery. After removing the doxycycline diet, animals were smeared daily in the morning between 09:30 and 10:30 for estrous cycle monitoring and again between 12:30 and 14:30 on proestrus to confirm the staging. Mice in proestrus underwent tail-tip bleeding as reported previously (Czieselsky et al., 2016) with 3 µl samples taken every 30- or 60-min between 15:00 and 22:30. Mice were then anesthetized and perfusion-fixed at variable times between 18:00 and 22:30.

### LH ELISA

The LH level was assayed using an ultrasensitive ELISA designed by Steyn and colleagues (28) (sensitivity, 0.04 ng/ml; intra- and interassay coefficients of variation of 6.0 and 13.3%, respectively). Over the course of the study, we transitioned from rabbit polyclonal LH antibody (AFP240580Rb) detection antibody (Steyn et al., 2013) to mouse monoclonal LH antibody (5303 SPRN) that was recently validated by Kreisman and coworkers (Kreisman et al., 2022; sensitivity, 0.08 ng/ml; intra- and interassay coefficients of variation of 4.8 and 20.8%, respectively).

### Immunohistochemistry and imaging

Mice were anesthetized with pentobarbital (2 mg/kg body weight per 100 µl, i.p.) and perfused transcardially with 4% paraformaldehyde in 0.1 M phosphate-buffered saline (Sigma-Aldrich, pH 7.6). Coronal brain sections of 40 µm thickness were cut using a freezing microtome. Dual GnRH-RAM immunofluorescence was undertaken using GA2 guinea pig anti-GnRH antisera (Rizwan et al., 2009) and rabbit anti-red fluorescent protein (Table 1). The sections were incubated with the cocktail of primary antibodies for 48 h at 4°C. Following that, sections were incubated with Alexa Fluor 647–conjugated goat anti-guinea pig (1:500) and Alexa Fluor 568–conjugated goat anti-rabbit immunoglobulins (1:500) for 1.5 h at room temperature. Imaging was undertaken with a Leica SP8 confocal microscope and analyzed using ImageJ. For analyzing the duration of RAM expression within GnRH neurons, two OVLT sections were selected from each mouse. To examine the overall distribution of RAM+ GnRH neurons, two sections containing the medial septum (MS), diagonal band of Broca (DBB), OVLT, or medial preoptic area (mPOA) were analyzed from each animal.

**Table 1.** List of antibodies, reagents, mouse lines, and software with their corresponding RRIDs that were being used in this research

Reagent	Source	Identifier
Antibodies		
Rabbit anti-GnRH, LR5 1:20,000	R. Benoit, McGill University	AB_2314605
Guinea pig anti-GnRH, GA02 1:3,000	G. Anderson, University of Otago	AB_2721118
Rabbit anti–c-Fos, SC52 1:3,000	Santa Cruz Biotechnology	AB_2106783
Rabbit anti–c-Jun (60A8), 9,165 1:2,000	Cell Signaling Technology	AB_2130165
Rabbit anti-red fluorescent protein, 600-402-379 1:8,000	Rockland Immunochemicals	AB_828391
Goat biotinylated anti-rabbit, BA-1000 1:200	Vector Laboratories	AB_2313606
Alexa Fluor 568 goat anti-rabbit, A-11011 1:500	Thermo Fisher Scientific	AB_143157
Alexa Fluor 647 goat anti-guinea pig, A-21450 1:500	Thermo Fisher Scientific	AB_2735091
Bacterial and virus strains		
AAV1-RAM-d2tTA-pA:TRE-FLEX-TdTomato-WPRE-pA	Yingxi Lin, MIT	#84468-AAV1 RRID: Addgene_84468
Experimental models: organisms/strains		
*Mouse*: *Lhrh-cre* (Tg[Gnrh1-Cre]1Dlc)	The Jackson Laboratory	RRID:IMSR_JAX:021207
Mouse: B6.Cg-*Igs7*^*tm162.1(tetO-GCaMP6s,CAG-tTA2)Hze*^/J	The Jackson Laboratory	RRID:IMSR_JAX:031562
Software and algorithms		
ImageJ	Schneider, et al.	https://imagej.nih.gov/ij/RRID: SCR_003070
Open-source NeuroMouse fiber photometry data acquisition system	Tussock Innovation and Agrotech	https://www.otago.ac.nz/neuroendocrinology/reources/argotech.html
LAS X (Leica Application Suite) confocal microscopy software	Leica Microsystems	https://www.leica-microsystems.com/RRID: SCR_013673

For dual GnRH and c-Fos/c-Jun immunohistochemistry, sections underwent peroxidase blocking for 15 min and were then washed and incubated for 48 h at 4°C with either rabbit anti–c-Fos or rabbit anti–c-Jun primary antibodies (Table 1). The sections were then incubated with biotinylated goat anti-rabbit immunoglobulins, followed by a 1:200 ABC peroxidase kit, and then reacted with nickel DAB. Sections were then incubated with guinea pig anti-GnRH GA2 for 48 h at 4°C followed by Alexa Fluor 647–conjugated goat anti-guinea pig immunoglobulins (Table 1) for 1.5 h. Sections were washed, mounted on slides, and imaged immediately. The specificity of both the c-Fos and c-Jun antisera have been reported previously (Clarkson et al., 2008; Matsuoka et al., 2010). Omitting any of the primary antisera from the incubation medium resulted in an absence of relevant immunoreactivity. Brightfield and fluorescence images were taken using an Olympus BX43 epifluorescence microscope. For each animal, two sections from each of the OVLT and mPOA areas were selected, and the total number of GnRH neurons and the number of GnRH neurons with either c-Fos or c-Jun were counted.

To establish the threshold for c-Fos and c-Jun nickel DAB staining, eight brightfield images representing the MS, DBB, OVLT, and mPOA regions (two sections per region) from each animal were converted to 8-bit format using ImageJ, with thresholds adjusted accordingly. The mean minus 2× SD for all values was 88 gray units, and, as such, this was used as a threshold for detecting a positive signal and applied in all subsequent analyses. In dual- and triple-labeled experiments, a GnRH neuron was considered to be positive if c-Fos or c-Jun immunoreactivity was restricted to the nuclear compartment of the cell and, where necessary, *Z*-plane focussing used to ensure that the immunoreactive nucleus was within and not below or above the GnRH neuron.

For triple GnRH-RAM-c-Fos/c-Jun immunohistochemistry, the immunolabeling was performed as noted above with the addition of rabbit anti-red fluorescent protein antisera to the second immunostaining step. Imaging was undertaken immediately using an Olympus BX43 epifluorescence microscope. For each animal, two sections from each of the OVLT and mPOA areas were selected, and the total number of GnRH neurons and the number of GnRH neurons with RAM, c-Fos, or c-Jun were counted. In all cases, sections were analyzed in a blinded manner.

### Statistical analysis

All statistical analyses were performed in Prism 10 (GraphPad Software). All values given in this study are mean ± SEM, and significance is defined as *p* < 0.05*, *p* < 0.01**, or *p* < 0.001***. For cell counts involving GnRH neurons, Shapiro–Wilk and Kolmogorov–Smirnov normality tests were conducted to assess whether the data followed a normal distribution. When the data did not meet the normality assumptions, Kruskal–Wallis nonparametric tests were performed instead.

## Results

### Variable patterns of GnRH neuron activity and LH secretion occur across successive proestrous surges in the same mice

**Figure 1.**
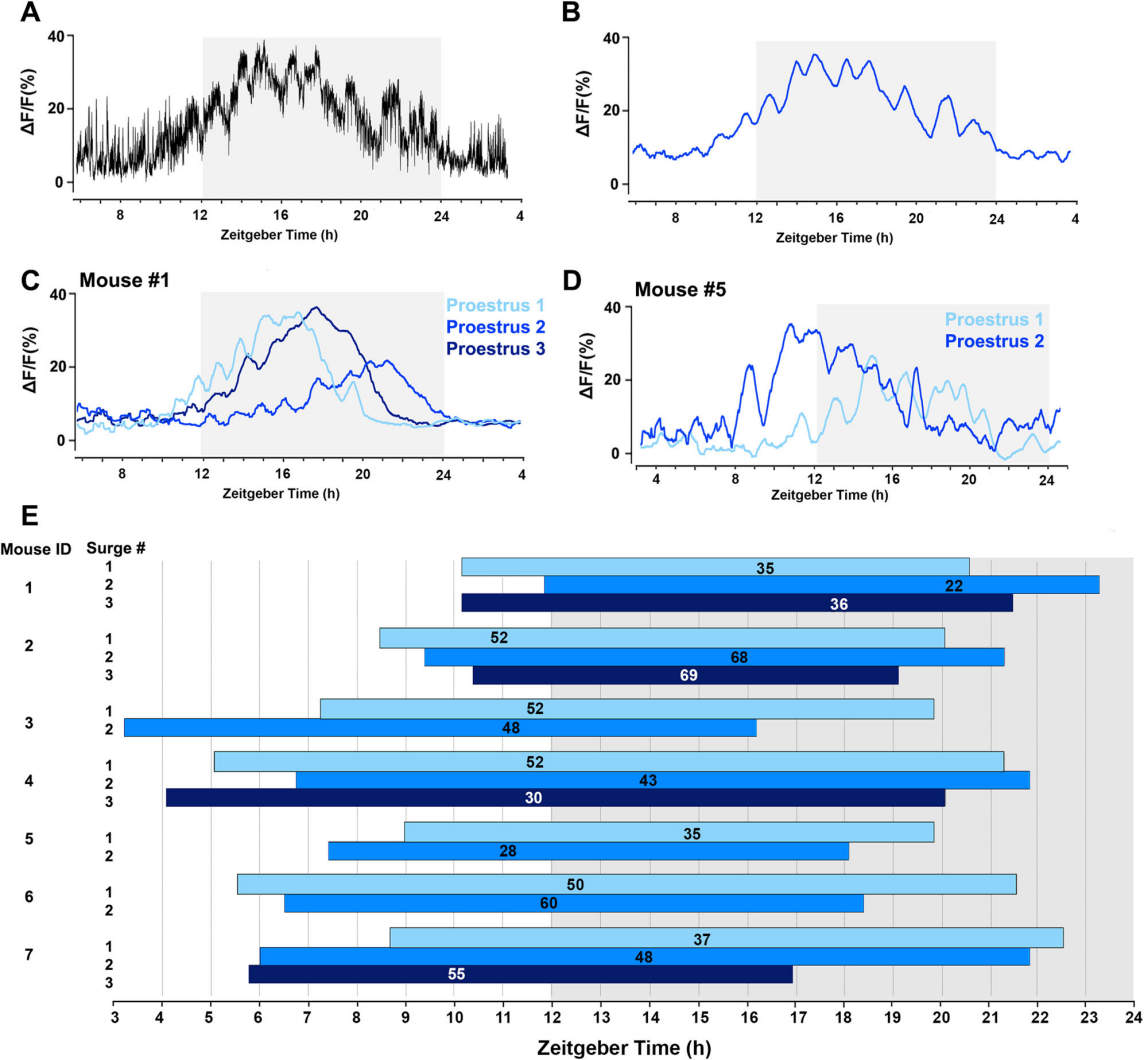
Variable patterns of GnRH neuron dendron activity across successive proestrous surges in the same mice. ***A***, Example raw trace of 21 h GnRH dendron GCaMP fiber photometry recording starting at Zeitgeber time 6:00 h (equals 1:00 P.M.). Lights off at Zeitgeber time 12:00 h. ***B***, Code-deconvoluted baseline profiles of GCaMP signals in GnRH neuron dendron activity of ***A*** demonstrating a clearer view of the onset and offset of the surge signal, consisting of multiple slow oscillations. ***C, D***, Two representative code-extracted baseline profiles of GCaMP signals in GnRH neuron dendron activity across two or three successive proestrous surges in two mice. ***E***, Chart displaying parameters of surge activity in GnRH neurons from successive proestrous days in seven mice. The horizontal blue-shaded bars for each mouse show the onset and offset of surge activity. Lights-off is indicated by the grey shaded area. The position of the number represents the time of peak GCaMP signal and the value shows the amplitude (DF/F%).

We previously observed that during proestrus, GnRH neuron dendrons exhibit a slow and large shift in baseline GCaMP activity consisting of multiple slow oscillations that correspond to, and greatly outlast, the LH surge (Han et al., 2024). To examine the consistency of this surge-like behavior, we made 24 h recordings of GnRH neuron dendron activity across two or three proestrous stages in seven mice. As expected, a gradual increase in baseline activity was observed to begin on the afternoon of proestrus (Fig. 1*A*). To help clarify the dynamics of the baseline shift in GCaMP activity occurring on proestrus, we used a customized Matlab code (a 30 min rolling average) (Han et al., 2024) to extract the slow oscillatory rise and decline in GCaMP activity (Fig. 1*B*). The slow increment in baseline calcium signal was not smooth but consisted of multiple slow oscillations (Fig. 1*A,B*) with a mean duration of 78 ± 4 min (range, 32–150 min;  *N* = 7 mice). We found a very wide range in the onset of surge activity between mice. While, on average, the baseline started to shift 4.9 ± 0.8 h (*N* = 7 mice) before the “lights off” (12 h:12 h, lights on at 7 A.M.), this could occur from 8.0 to 1.4 h before lights off for individual mice. This baseline rise peaked at 5.9 ± 0.5 h after beginning and then subsided so as to be completed 13.0 ± 0.6 h later (range, 8.8–16.6 h; Fig. 1*A–D*). Unexpectedly, when examining surge activity in the same mice, we found that all seven individual mice exhibited differences in the time of surge onset (1.0 ± 0.2 h difference; range, 0.8–4.1 h, *N* = 7 mice), maximum GCaMP signal amplitude (16 ± 2.9% difference; range, 4–27%,  *N* = 7 mice), and duration (2.1 ± 0.5 h difference; range, 0.05–4.5 h,  *N* = 7 mice) of dendron activity across subsequent proestrous surges (Fig. 1*C–E*). There did not appear to be any consistent variation between surge onsets with some mice showing progressively sooner or later surges while others fluctuated backwards and forwards (Fig. 1*E*).

**Figure 2.**
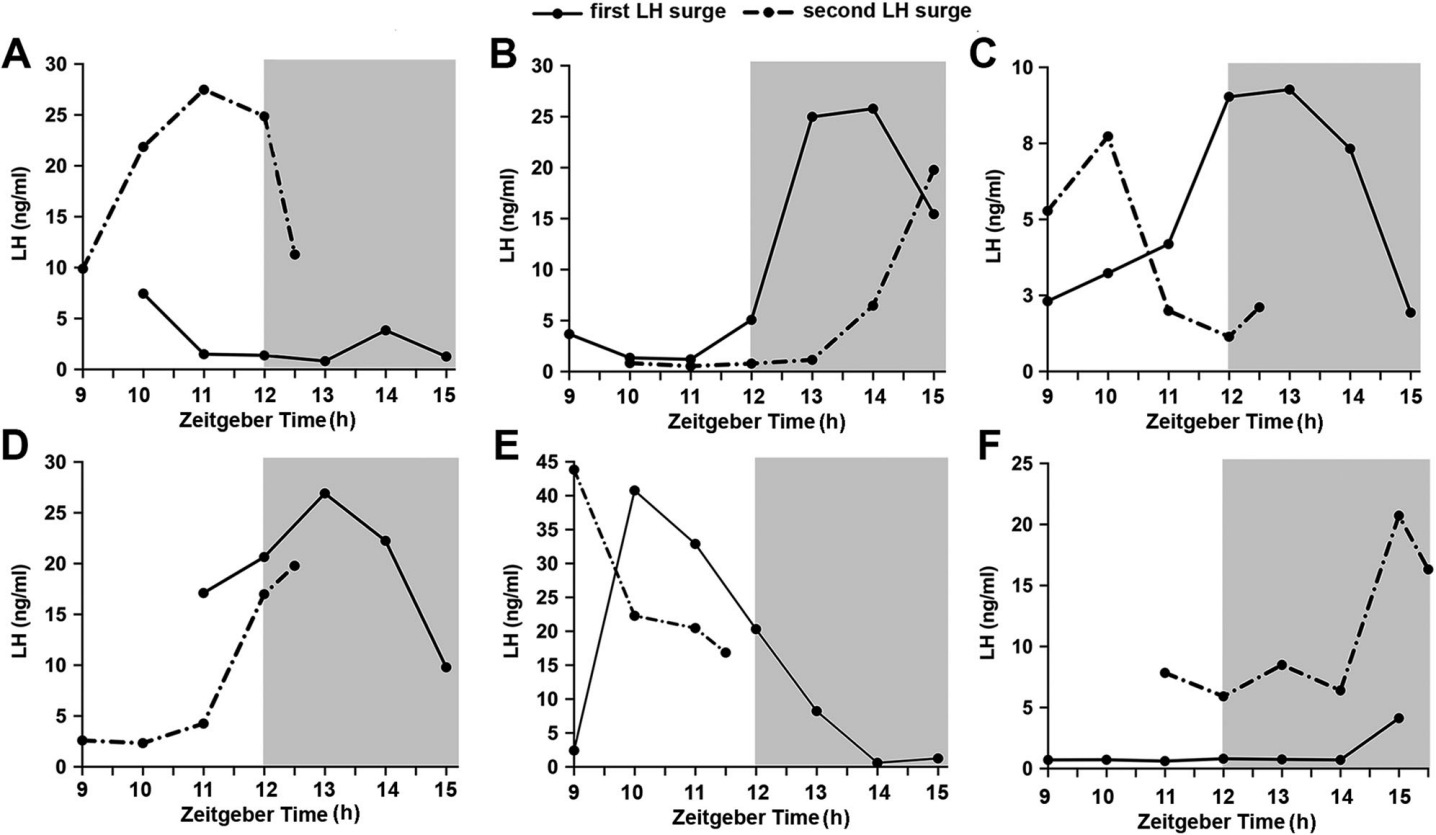
Variable patterns of LH surges across successive proestrous surges in the same mice. Graphs show LH levels of six proestrous mice (***A–F***) undergoing hourly tail-tip bleeding for 4–5 h on successive proestrous days. The gray shading indicates lights-off period starting at 7:00 P.M. represented as Zeitgeber time 12:00 h.

To examine whether variability exists in the onset of subsequent LH surges in the same mice, we undertook hourly tail-tip bleeding across two successive proestrous surges in six well-habituated mice. The temporal resolution afforded by blood sampling is poor compared with photometry, but the onsets of each surge can be approximated by the ascending and descending profiles of LH secretion at each surge. In this case, we observed some mice in which the timing of the surge varied by at least 2 h across consecutive proestrous surges (Fig. 2*A–C*). As far as could be ascertained, other mice had surge onsets or peaks occurring within an hour of each other (Fig. 2*D–F*).

### Immediate early gene expression by GnRH neurons at the proestrous LH surge

Together the observations above indicate that the surge mechanism can be labile with different onset times and profiles in the same mouse. We therefore questioned whether there may be different patterns of GnRH neuron activation occurring across successive surges in the same mouse. To explore this, we took advantage of genetic tools that allow activity tagging over extended periods of time and combined these with c-Fos/c-Jun immunohistochemistry that provides an assessment of very recent neuronal activation.

Early studies in rats had demonstrated that both c-Fos and c-Jun are expressed by GnRH neurons at the time of the surge with expression of the latter being more persistent (Lee et al., 1992). To assess c-Fos and c-Jun expression in mice, we examined the profile of immediate early gene protein expression in GnRH neurons during the proestrous surge. Starting between 14:00 and 16:00 on proestrous afternoon, hourly tail-tip blood samples were taken over four hours at which time the mouse was anesthetized and perfusion-fixed for dual GnRH/c-Fos and GnRH/c-Jun immunohistochemistry. A group of three diestrous-stage mice were examined at the same time. Depending on the profile of LH secretion, mice were then classified as having been killed 0–2 h (*N* = 6) or 2–4 h (*N* = 6) after the peak of the LH surge.

**Figure 3.**
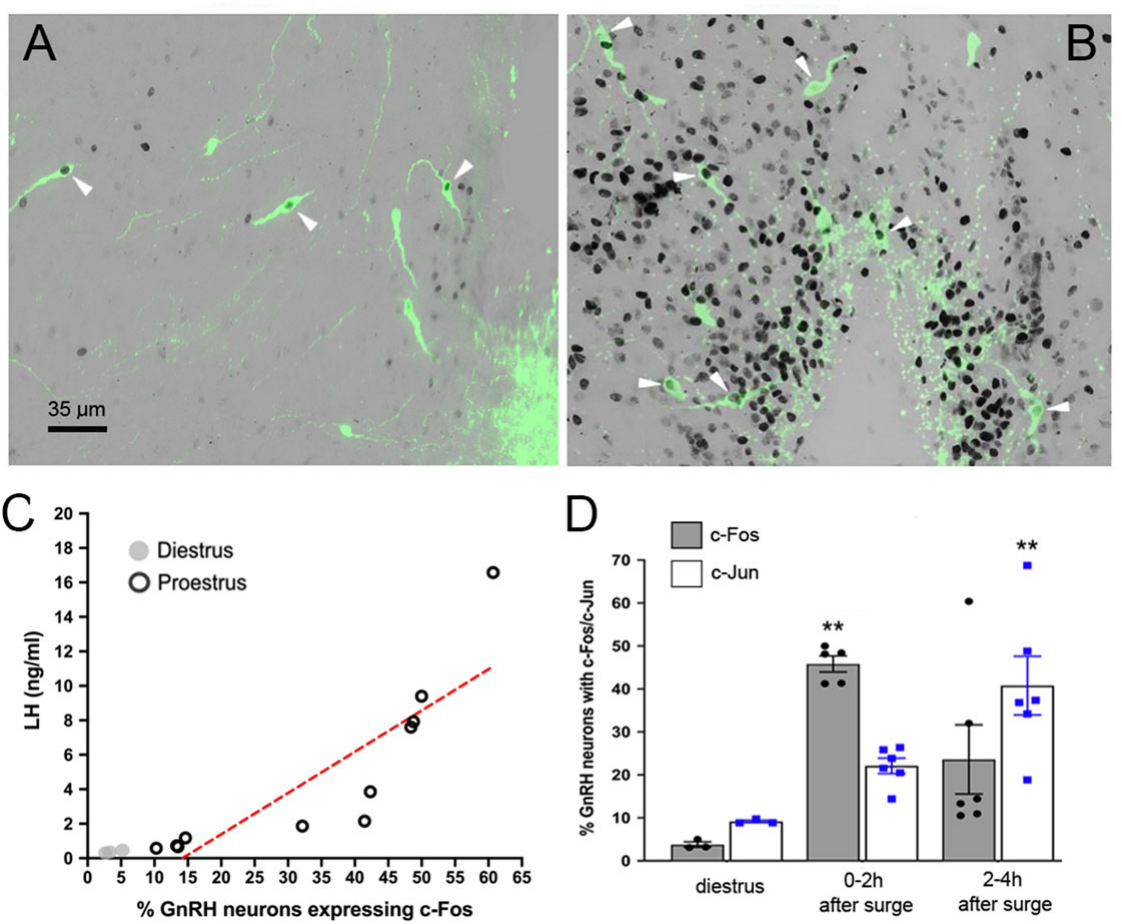
Expression of c-Fos and c-Jun in GnRH neurons at the time of the proestrous surge. ***A***, Image depicting nuclear-located c-Fos expression (black) in GnRH neurons (green) within the organum vasculosum of the lamina terminalis (OVLT) of a mouse killed 0–2 h after the peak of the LH surge. ***B***, Image depicting nuclear-located c-Jun expression (black) in OVLT GnRH neurons (green) of a mouse killed 2–4 h after the peak of the LH surge. ***C***, Graph showing the correlation between LH levels and percentage of GnRH neurons expressing c-Fos in diestrous and proestrous mice (*N* = 11). ***D***, Histogram showing the proportion of GnRH neurons expressing c-Fos and c-Jun in diestrous control (*N* = 3) and proestrous mice culled 0–2 h (*N* = 5) and 2–4 h (*N* = 6) relative to the peak of the LH surge. **p < 0.001 compared with diestrus, Kruskal–Wallis with Dunn's multiple-comparisons test.

Immunohistochemistry revealed the typical scattered distribution of c-Fos immunoreactive cells in the rPOA including within many GnRH neurons (Fig. 3*A*). The density of c-Jun–immunoreactive cells was substantially greater and also included many double-labeled neurons (Fig. 3*B*). Very few GnRH neurons expressed c-Fos (4%) or c-Jun (9%) in diestrous mice (Fig. 3*D*). For c-Fos, there was a significant increase in GnRH neurons expressing c-Fos in mice killed 0–2 h after the peak of the LH surge (46.2 ± 1.8%, *N* = 5 as one set of sections was lost) compared with diestrus (*p* = 0.0096, Kruskal–Wallis with Dunn's multiple-comparisons test), and this was reduced to 23.7 ± 8.1% in the 2–4 h time period (*N* = 6; Fig. 3*D*). When all proestrous mice with c-Fos data were considered together, there was a strong correlation between the peak LH surge levels and the % of GnRH neurons expressing c-Fos (*N* = 11, *R*^2^ = 0.74, *F*_(1,12)_ = 34, *p* < 0.001, simple linear regression; Fig. 3*C*). For c-Jun, 22.0 ± 1.8% of GnRH neurons expressed c-Jun in the 0–2 h group, and compared with diestrus, this increased significantly to 40.7 ± 6.8% in the 2–4 h time period (*p* = 0.0067, Kruskal–Wallis with Dunn's multiple-comparisons test; Fig. 3*D*). The numbers of GnRH neurons detected were not different between any of the groups (c-Fos, diestrus 34 ± 3, 0–2 h 34 ± 3, 2–4 h 34 ± 3; c-Jun, diestrus 40 ± 3, 0–2 h 40 ± 2, 2–4 h 38 ± 4 GnRH neurons/section). Together, this indicated that c-Fos was a good marker of activated GnRH neurons when mice were killed within 2 h of peak LH surge levels whereas c-Jun was a better indicator of surge-activated GnRH neurons at later time points.

### Long-term labeling of surge-activated GnRH neurons

**Figure 4.**
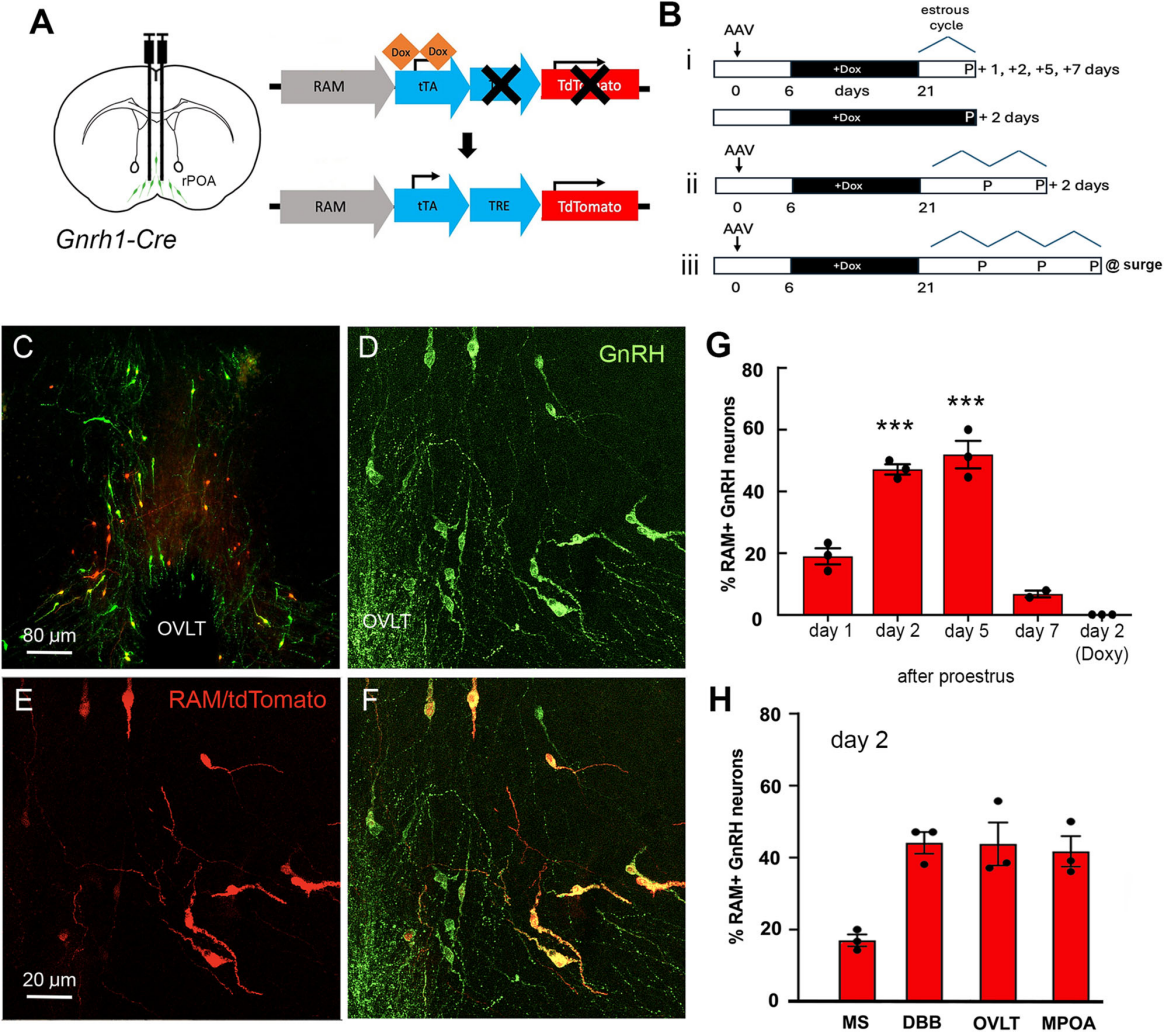
Long-term labeling of activated GnRH neurons with robust activity marking (RAM) strategy. ***A***, Schematic showing strategy of bilateral injection of cre-dependent AAV1-RAM into the rPOA of *Gnrh1-Cre* mice and the doxycycline-dependent RAM strategy. ***B***, Animal treatment timelines. +Dox, mice on doxycyline diet; P, proestrous stage of cycle. ***C***, A low-power image of dual label fluorescence for GnRH (green) and RAM-tdTomato (red) in the rostral preoptic area of an AAV-RAM, *Gnrh1-Cre* female mouse killed 2 d after proestrus. ***D–F***, Higher-power images showing the expression of RAM-tdTomato (red) in several GnRH neurons (yellow) around the organum vasculosum of the lamina terminalis (OVLT). ***G***, Histogram showing the percentage of rPOA GnRH neurons expressing RAM in animals that were culled at different time points after their first proestrus (***Bi***). To assess the efficacy of the doxycycline diet in inhibiting the RAM construct, one group of animals was treated continuously with the doxy diet and culled 2 d after proestrus. ***H***, Histogram showing the distribution of RAM+ GnRH neurons in the medial septum (MS), diagonal band of Broca (DBB), OVLT, and the medial preoptic area (mPOA) of mice killed 2 d after their second consecutive proestrus in the absence of doxycycline (***Bii***). ***p < 0.0001 compared with Day 1, one-way ANOVA post hoc Dunnett's multiple comparison.

To achieve a longer-lasting record of GnRH neurons activated at the time of the surge, we used a genetically encodable robust activity marking (RAM) approach in which a Cre- and doxycycline Tet-off–dependent synthetic neuronal activity promoter drives the expression of tdTomato (Sorensen et al., 2016; Fig. 4*A*). This system enables cell-specific reporting of activated neurons within a controlled time window.

We first examined the temporal dynamics of tdTomato expression in GnRH neurons following their activation on proestrus. Six days after bilateral injections of AAV1-RAM-d2tTA-pA::TRE-TdTomato into the rPOA, GnRH1-Cre mice were placed on a doxycycline chow diet (Fig. 4*A,B*). Two weeks later, mice were returned to their normal chow, and estrous cycles were monitored so that mice were killed 1 or 2 d after their first day of proestrus (*N* = 3, each). Mice exhibiting estrous cycles greater than 4 d in length were killed at 5 (*N* = 3) or 7 (*N* = 2) days after their first proestrus (Fig. 4*Bi*) to assess how quickly tdTomato degrades. One group of mice (*N* = 3) was maintained on the doxycycline chow diet for the duration of the experiment and killed 2 d after proestrus.

Mice with bilateral AAV injections in the rPOA were found to exhibit tdTomato expression in GnRH neurons (Fig. 4*C–F*) in a manner dependent on time after proestrus (Fig. 4*G*). One day after proestrus, 19.0 ± 2.6% of GnRH neurons were labeled with tdTomato, and this increased to 47.4 ± 1.5% on Day 2 (one-way ANOVA *p* < 0.0001, *F* = 47.36; *p* = 0.0006, post hoc Dunnett's multiple comparison) and 52.1 ± 4.6% on Day 5 (*p* = 0.003, post hoc Dunnett's test; Fig. 4*G*). Mice killed 7 d after proestrus exhibited few tdTomato-expressing GnRH neurons (6.8 ± 1.0%; *p* = 0.0825, post hoc Dunnett's test). The GnRH neurons of mice remaining on the doxycycline diet throughout the protocol failed to exhibit any tdTomato expression (Fig. 4*G*). The total numbers of GnRH neurons analyzed were not different in each treatment group (Day 1, 26 ± 5; Day 2, 23 ± 2; Day 5, 24 ± 3; Day 7, 21 ± 4 GnRH neurons within AAV injection site/section). This indicates that, as found for other neuronal populations (Sorensen et al., 2016), RAM-dependent expression of tdTomato in GnRH neurons is maximal 2–5 d after intense activation and very effectively controlled by doxycycline.

We next examined the effect of allowing the RAM system to label GnRH neurons across two consecutive surges. Mice (*N* = 3) were treated in the same manner above except that they were killed 2 d after proestrus of their second estrous cycle in the absence of doxycycline (Fig. 4*Bii*). In these mice, we assessed activation across the rostrocaudal distribution of GnRH neurons by analyzing mice in which bilateral AAV injections encompassed the medial septum (MS), diagonal band of Broca (DBB), organum vasculosum of the lamina terminalis (OVLT), and/or the medial preoptic area (mPOA). Approximately 40–50% of GnRH neurons expressed tdTomato across the DBB, OVLT, and mPOA, while fewer activated GnRH neurons were detected in the MS (Fig. 4*H*). The overall percentage of GnRH neurons expressing tdTomato in mice was 37.3 ± 6.0%.

### Different overlapping GnRH neuron ensembles are active on successive proestrous surges

**Figure 5.**
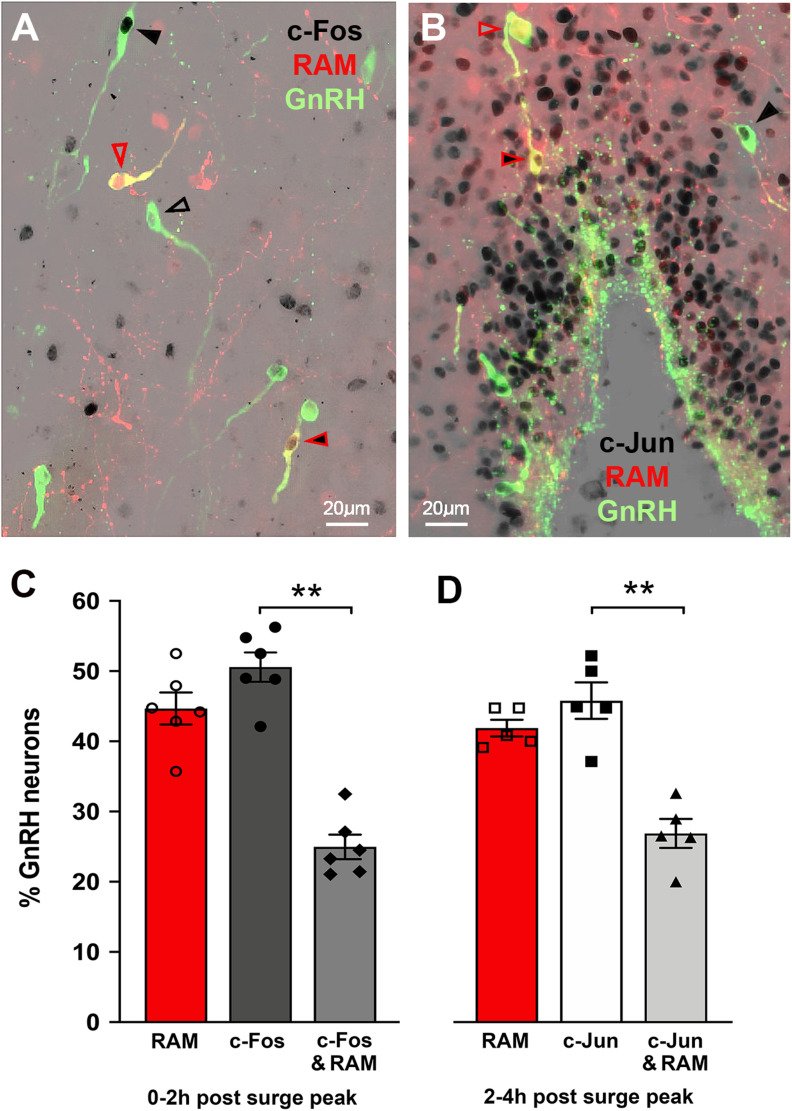
Different subpopulations of GnRH neurons are activated on successive proestrous surges. ***A, B***, Triple immunofluorescence labeling showing GnRH neurons (green) with and without black c-Fos (***A***) or c-Jun (***B***)-positive nuclei and RAM-positive GnRH neurons (yellow) with and without c-Fos/c-Jun–positive nuclei. Fully black arrowheads, GnRH neurons with c-Fos/c-Jun only; black outline arrowheads, GnRH neurons without c-Fos; red outline arrowheads, GnRH neurons with RAM only; red arrowheads with black, GnRH neurons expressing RAM and c-Fos/c-Jun. ***C***, Histogram showing the percentage of all rPOA GnRH neurons expressing RAM, c-Fos, and both RAM and c-Fos in mice killed 0–2 h after the peak of the LH surge (*N* = 6). ***D***, Histogram showing the percentage of all rPOA GnRH neurons expressing RAM, c-Jun, and both RAM and c-Jun in mice killed 2–4 h after the peak of the LH surge (*N* = 5). **p < 0.01 Kruskal–Wallis test, Dunn's multiple-comparisons test.

We next combined the c-Fos/c-Jun and RAM approaches to examine the relationship between GnRH neurons activated during the current (expressing c-Fos or c-Jun) and prior GnRH surge (expressing tdTomato). *Gnrh1*-Cre mice were given bilateral AAV injections targeting the rPOA/OVLT region and doxycycline treatment as above and then, to ensure the maximal activation history was captured, killed on the evening of proestrus after two prior surges (Fig. 4*Biii*). Due to the considerable range in the timing of surge onset (Fig. 2), we returned to our protocol of taking hourly tail-tip blood samples so that we could retrospectively group mice into those killed within 2 h of peak LH surge levels and those killed 2–4 h after the peak of the surge. We used c-Fos immunohistochemistry to determine GnRH neurons activated in the 0–2 h group (*N* = 6, peak LH levels 18.9 ± 2.8 ng/ml) and c-Jun in the 2–4 h group (*N* = 5, peak LH levels 26.0 ± 5.8 ng/ml; Fig. 3*D*). In both groups, we observed GnRH neurons expressing c-Fos/c-Jun as well as tdTomato, in addition to GnRH neurons with either c-Fos/c-Jun or tdTomato, and GnRH neurons that had no activity markers (Fig. 5*A,B*). Importantly, rPOA GnRH neurons expressing different combinations of c-Fos/c-Jun and tdTomato were intermingled (Fig. 5) indicating that those without tdTomato did not do so because they had not been transfected with the AAV. In the 0–2 h group, 50.6 ± 2.1% of all GnRH neurons were immunoreactive for c-Fos, and 44.7 ± 2.3% of all GnRH neurons expressed tdTomato. However, only approximately half of c-Fos–positive GnRH neurons, representing 25.0 ± 1.8% of all GnRH neurons, also expressed tdTomato (*p* = 0.0015, *Z* = 3.49, Kruskal–Wallis test, Dunn's multiple-comparisons test; Fig. 5*C*). Similarly, in the 2–4 h group, 41.9 ± 1.2% of all GnRH neurons expressed tdTomato, 45.8 ± 2.6% of all GnRH neurons were immunoreactive for c-Jun, but only 26.9 ± 2.1% of all GnRH neurons had both markers of IEG activation (*p* = 0.0054, *Z* = 3.12, Kruskal–Wallis test, Dunn's multiple-comparisons test; Fig. 5*D*). The total numbers of GnRH-immunoreactive neurons analyzed were not different across groups (c-Fos, 43 ± 2; c-Jun, 41 ± 3 GnRH neurons/section). This indicates that only approximately half of all activated rPOA GnRH neurons contributing to a proestrous surge were involved in the previous two surges.

## Discussion

The remote monitoring of the GnRH neuron ensemble in freely behaving mice has allowed us to examine the consistency of the surge mechanism without the need for blood sampling or other significant human intervention. This has confirmed the marked variability in the timing of the LH surge observed previously in mice (Miller et al., 2004; Minabe et al., 2011; Czieselsky et al., 2016) and indicates that this inconsistency is an authentic biological variable rather than an experimentally induced phenomenon. Unexpectedly, however, we found that this variability extended to the proestrous surges exhibited by individual animals with some mice exhibiting up to a 4 h time difference in surge onset during consecutive estrous cycles. Even more surprising, the combination of conventional and novel genetic activation markers has revealed that different GnRH neurons join the ensemble responsible for triggering preovulatory surges on consecutive cycles.

The seminal observation that c-Fos could be used to mark GnRH neurons involved in the GnRH surge (Lee et al., 1990) provided an important immunohistochemical window for examining their nature and characteristics (Hoffman et al., 1993; Herbison, 2015). This was proven possible in several species with approximately 50% of GnRH neurons identified to express c-Fos shortly after the onset of the estrogen- or mating-induced LH surge in mice (Wintermantel et al., 2006), rats (Lee et al., 1990), ferrets (Lambert et al., 1992), rabbits (Caba et al., 2000), and sheep (Moenter et al., 1993), but curiously not monkeys (Witkin et al., 1994). The c-Fos–expressing subpopulation of GnRH neurons in rodents has been explored in detail and reported to be more transcriptionally active, receive different patterns of innervation, and also be more likely to coexpress galanin (for review, see Herbison, 2015). Together, these observations fostered the notion that a specific subpopulation of “surge GnRH neurons” existed alongside others with, albeit, ill-defined functions (Herbison, 2015).

The present study suggests that the great majority, and possibly all, rPOA GnRH neurons are involved in surge generation but not on the same day. We find that approximately 50% of GnRH are recruited to drive each GnRH surge at any one time but that only half of these cells were active in the prior surge. This implies that at least 75% of GnRH neurons can operate as “surge GnRH neurons,” and, although beyond our technical capability, it is conceivable that all rPOA GnRH neurons are involved in surge generation at some point. This would be compatible with the observation that over 90% of GnRH neurons express *Kiss1r* and have their cell bodies activated by kisspeptin (Han et al., 2005), the key output of the rostral periventricular area of the third ventricle (RP3V) surge generator (Clarkson et al., 2023). As such, it is likely that there is no special subpopulation of GnRH neurons dedicated to the LH surge or the generation of LH pulses. Indeed, the two-compartment model of GnRH neuron function (Herbison, 2020) has the potential for all GnRH neurons to be involved in surge generation at the level of their cell bodies while also contributing to pulses at their distal projections (Wang et al., 2020; Herbison, 2021; Goodman et al., 2022).

The mechanism underlying this remarkable network plasticity is unknown. Interestingly, it has been suggested that neurons within a network may actually compete for inclusion in neuronal ensembles underlying aspects of memory (Josselyn and Tonegawa, 2020). The intermittent recruitment of individual GnRH neurons to the surge ensemble may result from stochasticity within the GnRH neuron population itself. Equally, given the importance of neural inputs conveying information on circulating estradiol and circadian status for surge generation (Goodman et al., 2022), shifting GnRH neuron ensembles may result from variations in these afferent inputs.

Considering the first possibility, it is readily apparent that at any one experimental instance, substantial heterogeneity exists in the ion channels, neurotransmitter receptors, and firing behavior exhibited by GnRH neurons (Herbison, 2015; Spergel, 2019; Constantin et al., 2021). Thus, it may be the case that the circadian and kisspeptin surge generator output is relayed in a relatively homogeneous and consistent manner to GnRH neurons but that ongoing levels of excitability and synaptic integration determine whether an individual GnRH neuron participates in the forthcoming surge. Many different neurotransmitters and neuropeptides have been found to directly inhibit the activity of subpopulations of GnRH neurons (Herbison, 2015; Spergel, 2019) and these may variably counter the typically strong excitation provided by kisspeptin.

It is also possible that the RP3V kisspeptin surge generator, integrating multiple cues for surge initiation (Goodman et al., 2022; Kauffman, 2022; Piet, 2023), provides a variable excitatory input to different GnRH neurons on proestrus. Studies using c-Fos as an indicator suggest that only approximately 30–40% of the RP3V kisspeptin neuron population is activated at the time of the surge (Clarkson et al., 2008; Robertson et al., 2009). However, this estimate requires studies similar to those performed here to establish whether there is also cycle-to-cycle plasticity in RP3V kisspeptin neuron activation. While it is clear that RP3V kisspeptin neurons innervate and form direct synapses with rPOA GnRH neuron somata (Yip et al., 2015; Piet et al., 2018; Liu et al., 2021), the precise innervation patterns of GnRH neuron cell bodies by RP3V kisspeptin neurons, including their levels of divergence, are unknown.

Finally, it is worth considering the possibility that cycle-to-cycle plasticity may exist in the direct suprachiasmatic nucleus (SCN) vasoactive intestinal peptide (VIP) input to GnRH neuron somata. The LH surge is one of many time-locked circadian events, and, in addition to an arginine vasopressin input to the RP3V kisspeptin neurons, a direct VIP projection exists to a subpopulation of GnRH neurons (Tonsfeldt et al., 2022; Piet, 2023). Interestingly, a preferential VIP innervation of c-Fos–expressing GnRH neurons at the time of the surge has been reported in rats (van der Beek et al., 1994). Furthermore, the suppression of VIP transmission results in altered timing of the surge alongside a reduction in the numbers of c-Fos–activated GnRH neurons (van der Beek et al., 1999; Gerhold et al., 2005). However, a GCaMP fiber photometry study has shown that changes in SCN VIP neuron activity do not align closely with the onset of the LH surge with activity declining abruptly at lights out (Jones et al., 2018). This may make it difficult to ascribe any sustained activational role for SCN VIP inputs to GnRH neurons at this time.

In conclusion, the observation here of shifting GnRH neuron ensembles responsible for consecutive LH surges indicates the existence of a unique pattern of oscillating network plasticity within the normal physiological mechanism responsible for the surge. From one perspective, this is likely to contribute to variability in the dynamics of the surge from cycle to cycle and between individuals. Indeed, as found for mice, preovulatory surge onset, amplitude, and dynamics are also highly variable in women (Park et al., 2007; Direito et al., 2013). However, any detrimental effects of variable surge timing for the individual may be outweighed by safeguarding the surge mechanism itself. Alongside evidence that only approximately 10% of the normal GnRH surge is required for ovulation (Karsch et al., 1997), the finding that shifting GnRH neuron ensembles are responsible for consecutive surges likely engenders considerable robustness to the mammalian ovulatory mechanism.

## References

[B1] Caba M, Pau KY, Beyer C, Gonzalez A, Silver R, Spies HG (2000) Coitus-induced activation of c-fos and gonadotropin-releasing hormone in hypothalamic neurons in female rabbits. Brain Res Mol Brain Res 78:69–79. 10.1016/S0169-328X(00)00071-1 10891586

[B2] Clarkson J, d’Anglemont de Tassigny X, Moreno AS, Colledge WH, Herbison AE (2008) Kisspeptin-GPR54 signaling is essential for preovulatory gonadotropin-releasing hormone neuron activation and the luteinizing hormone surge. J Neurosci 28:8691–8697. 10.1523/JNEUROSCI.1775-08.2008 18753370 PMC6670827

[B3] Clarkson J, Yip SH, Porteous R, Kauff A, Heather AK, Herbison AE (2023) CRISPR-Cas9 knockdown of ESR1 in preoptic GABA-kisspeptin neurons suppresses the preovulatory surge and estrous cycles in female mice. eLife 12:RP90959. 10.7554/eLife.90959.3 38126277 PMC10735218

[B4] Constantin S, Moenter SM, Piet R (2021) The electrophysiologic properties of gonadotropin-releasing hormone neurons. J Neuroendocrinol 34:e13073. 10.1111/jne.13073 34939256 PMC9163209

[B5] Czieselsky K, Prescott M, Porteous R, Campos P, Clarkson J, Steyn FJ, Campbell RE, Herbison AE (2016) Pulse and surge profiles of luteinizing hormone secretion in the mouse. Endocrinology 157:4794–4802. 10.1210/en.2016-1351 27715255

[B6] Daigle TL, et al. (2018) A suite of transgenic driver and reporter mouse lines with enhanced brain-cell-type targeting and functionality. Cell 174:465–480 e422. 10.1016/j.cell.2018.06.035 30007418 PMC6086366

[B7] Direito A, Bailly S, Mariani A, Ecochard R (2013) Relationships between the luteinizing hormone surge and other characteristics of the menstrual cycle in normally ovulating women. Fertil Steril 99:279–285 e273. 10.1016/j.fertnstert.2012.08.047 22999798

[B8] Duittoz AH, Forni PE, Giacobini P, Golan M, Mollard P, Negron AL, Radovick S, Wray S (2022) Development of the gonadotropin-releasing hormone system. J Neuroendocrinol 34:e13087. 10.1111/jne.13087 35067985 PMC9286803

[B9] Gerhold LM, Rosewell KL, Wise PM (2005) Suppression of vasoactive intestinal polypeptide in the suprachiasmatic nucleus leads to aging-like alterations in cAMP rhythms and activation of gonadotropin-releasing hormone neurons. J Neurosci 25:62–67. 10.1523/JNEUROSCI.3598-04.2005 15634767 PMC6725194

[B10] Goodman RL, Herbison AE, Lehman MN, Navarro VM (2022) Neuroendocrine control of gonadotropin-releasing hormone: pulsatile and surge modes of secretion. J Neuroendocrinol 34:e13094. 10.1111/jne.13094 35107859 PMC9948945

[B11] Han SK, Gottsch ML, Lee KJ, Popa SM, Smith JT, Jakawich SK, Clifton DK, Steiner RA, Herbison AE (2005) Activation of gonadotropin-releasing hormone neurons by kisspeptin as a neuroendocrine switch for the onset of puberty. J Neurosci 25:11349–11356. 10.1523/JNEUROSCI.3328-05.2005 16339030 PMC6725899

[B12] Han SY, Kane G, Cheong I, Herbison AE (2019) Characterization of GnRH pulse generator activity in male mice using GCaMP fiber photometry. Endocrinology 160:557–567. 10.1210/en.2018-01047 30649269

[B13] Han SY, Morris PG, Kim JC, Guru S, Pardo-Navarro M, Yeo SH, McQuillan HJ, Herbison AE (2023) Mechanism of kisspeptin neuron synchronization for pulsatile hormone secretion in male mice. Cell Rep 42:111914. 10.1016/j.celrep.2022.111914 36640343 PMC7618808

[B14] Han SY, Yeo S-H, Kim J-C, Zhou Z, Herbison AE (2024) Multi-dimensional oscillatory activity of mouse GnRH neurons in vivo. BioRxiv 2024.06.26.600804.

[B15] Herbison AE (2015) Physiology of the adult GnRH neuronal network. In: Knobil and Neill’s physiology of reproduction (Plant TM, Zeleznik AJ, eds), pp 399–467. San Diego: Academic Press.

[B16] Herbison AE (2020) A simple model of estrous cycle negative and positive feedback regulation of GnRH secretion. Front Neuroendocrinol 57:100837. 10.1016/j.yfrne.2020.100837 32240664

[B17] Herbison AE (2021) The dendron and episodic neuropeptide release. J Neuroendocrinol 33:e13024. 10.1111/jne.13024 34427000

[B18] Hoffman GE, Smith MS, Verbalis JG (1993) c-Fos and related immediate early gene products as markers of activity in neuroendocrine systems. Front Neuroendocrinol 14:173–213. 10.1006/frne.1993.1006 8349003

[B19] Jones JR, Simon T, Lones L, Herzog ED (2018) SCN VIP neurons are essential for normal light-mediated resetting of the circadian system. J Neurosci 38:7986–7995. 10.1523/JNEUROSCI.1322-18.2018 30082421 PMC6596148

[B20] Josselyn SA, Tonegawa S (2020) Memory engrams: recalling the past and imagining the future. Science 367:eaaw4325. 10.1126/science.aaw4325 31896692 PMC7577560

[B21] Karsch FJ, Bowen JM, Caraty A, Evans NP, Moenter SM (1997) Gonadotropin-releasing hormone requirements for ovulation. Biol Reprod 56:303–309. 10.1095/biolreprod56.2.303 9116125

[B22] Kauffman AS (2022) Neuroendocrine mechanisms underlying estrogen positive feedback and the LH surge. Front Neurosci 16:953252. 10.3389/fnins.2022.953252 35968365 PMC9364933

[B23] Kreisman MJ, McCosh RB, Breen KM (2022) A modified ultra-sensitive ELISA for measurement of LH in mice. Endocrinology 163:bqac109. 10.1210/endocr/bqac109 35869782 PMC9337274

[B24] Lambert GM, Rubin BS, Baum MJ (1992) Sex difference in the effect of mating on c-fos expression in luteinizing hormone-releasing hormone neurons of the ferret forebrain. Endocrinology 131:1473–1480. 10.1210/endo.131.3.1505478 1505478

[B25] Lee WS, Abbud R, Smith MS, Hoffman GE (1992) LHRH neurons express cJun protein during the proestrous surge of luteinizing hormone. Endocrinology 130:3101–3103. 10.1210/endo.130.5.1572316 1572316

[B26] Lee WS, Smith MS, Hoffman GE (1990) Luteinizing hormone-releasing hormone neurons express Fos protein during the proestrous surge of luteinizing hormone. Proc Natl Acad Sci U S A 87:5163–5167. 10.1073/pnas.87.13.5163 2114646 PMC54282

[B27] Lerner TN, et al. (2015) Intact-brain analyses reveal distinct information carried by SNc dopamine subcircuits. Cell 162:635–647. 10.1016/j.cell.2015.07.014 26232229 PMC4790813

[B28] Liu X, Yeo SH, McQuillan HJ, Herde MK, Hessler S, Cheong I, Porteous R, Herbison AE (2021) Highly redundant neuropeptide volume co-transmission underlying episodic activation of the GnRH neuron dendron. eLife 10:e62455. 10.7554/eLife.62455 33464205 PMC7847305

[B29] Matsuoka TA, Kaneto H, Miyatsuka T, Yamamoto T, Yamamoto K, Kato K, Shimomura I, Stein R, Matsuhisa M (2010) Regulation of MafA expression in pancreatic β-cells in db/db mice with diabetes. Diabetes 59:1709–1720. 10.2337/db08-0693 20424231 PMC2889771

[B30] Miller BH, Olson SL, Turek FW, Levine JE, Horton TH, Takahashi JS (2004) Circadian clock mutation disrupts estrous cyclicity and maintenance of pregnancy. Curr Biol 14:1367–1373. 10.1016/j.cub.2004.07.055 15296754 PMC3756147

[B31] Minabe S, Uenoyama Y, Tsukamura H, Maeda K (2011) Analysis of pulsatile and surge-like luteinizing hormone secretion with frequent blood sampling in female mice. J Reprod Dev 57:660–664. 10.1262/jrd.11-078S 21804302

[B32] Moenter SM, Karsch FJ, Lehman MN (1993) Fos expression during the estradiol-induced gonadotropin-releasing hormone (GnRH) surge of the ewe: induction in GnRH and other neurons. Endocrinology 133:896–903. 10.1210/endo.133.2.8344224 8344224

[B33] Park SJ, Goldsmith LT, Skurnick JH, Wojtczuk A, Weiss G (2007) Characteristics of the urinary luteinizing hormone surge in young ovulatory women. Fertil Steril 88:684–690. 10.1016/j.fertnstert.2007.01.045 17434509

[B34] Piet R (2023) Circadian and kisspeptin regulation of the preovulatory surge. Peptides 163:170981. 10.1016/j.peptides.2023.170981 36842628

[B35] Piet R, Kalil B, McLennan T, Porteous R, Czieselsky K, Herbison AE (2018) Dominant neuropeptide cotransmission in kisspeptin-GABA regulation of GnRH neuron firing driving ovulation. J Neurosci 38:6310–6322. 10.1523/JNEUROSCI.0658-18.2018 29899026 PMC6596098

[B36] Rizwan MZ, Porteous R, Herbison AE, Anderson GM (2009) Cells expressing RFamide-related peptide-1/3, the mammalian gonadotropin-inhibitory hormone orthologs, are not hypophysiotropic neuroendocrine neurons in the rat. Endocrinology 150:1413–1420. 10.1210/en.2008-1287 19008316

[B37] Robertson JL, Clifton DK, de la Iglesia HO, Steiner RA, Kauffman AS (2009) Circadian regulation of Kiss1 neurons: implications for timing the preovulatory gonadotropin-releasing hormone/luteinizing hormone surge. Endocrinology 150:3664–3671. 10.1210/en.2009-0247 19443569 PMC2717859

[B38] Sorensen AT, et al. (2016) A robust activity marking system for exploring active neuronal ensembles. eLife 5:e13918. 10.7554/eLife.13918 27661450 PMC5035142

[B39] Spergel DJ (2019) Modulation of gonadotropin-releasing hormone neuron activity and secretion in mice by non-peptide neurotransmitters, gasotransmitters, and gliotransmitters. Front Endocrinol 10:329. 10.3389/fendo.2019.00329 31178828 PMC6538683

[B40] Steyn FJ, Wan Y, Clarkson J, Veldhuis JD, Herbison AE, Chen C (2013) Development of a methodology for and assessment of pulsatile luteinizing hormone secretion in juvenile and adult male mice. Endocrinology 154:4939–4945. 10.1210/en.2013-1502 24092638 PMC5398599

[B41] Tonsfeldt KJ, Mellon PL, Hoffmann HM (2022) Circadian rhythms in the neuronal network timing the luteinizing hormone surge. Endocrinology 163:bqac268. 10.1210/endocr/bqab268 34967900 PMC8782605

[B42] van der Beek EM, Swarts HJ, Wiegant VM (1999) Central administration of antiserum to vasoactive intestinal peptide delays and reduces luteinizing hormone and prolactin surges in ovariectomized, estrogen-treated rats. Neuroendocrinology 69:227–237. 10.1159/000054423 10207274

[B43] van der Beek EM, van Oudheusden HJ, Buijs RM, van der Donk HA, van den Hurk R, Wiegant VM (1994) Preferential induction of c-fos immunoreactivity in vasoactive intestinal polypeptide-innervated gonadotropin-releasing hormone neurons during a steroid-induced luteinizing hormone surge in the female rat. Endocrinology 134:2636–2644. 8194489 10.1210/endo.134.6.8194489

[B44] Wagenmaker ER, Moenter SM (2017) Exposure to acute psychosocial stress disrupts the luteinizing hormone surge independent of estrous cycle alterations in female mice. Endocrinology 158:2593–2602. 10.1210/en.2017-00341 28549157 PMC5551545

[B45] Wang L, Guo W, Shen X, Yeo S, Long H, Wang Z, Lyu Q, Herbison AE, Kuang Y (2020) Different dendritic domains of the GnRH neuron underlie the pulse and surge modes of GnRH secretion in female mice. eLife 9:e53945. 10.7554/eLife.53945 32644040 PMC7347383

[B46] Wintermantel TM, et al. (2006) Definition of estrogen receptor pathway critical for estrogen positive feedback to gonadotropin-releasing hormone neurons and fertility. Neuron 52:271–280. 17046690 10.1016/j.neuron.2006.07.023 PMC6116893

[B47] Witkin JW, Xiao E, Popilskis S, Ferin M, Silverman A (1994) Fos expression in the gonadotropin-releasing hormone (GnRH) neuron does not increase during the ovarian steroid-induced GnRH surge in the rhesus monkey. Endocrinology 135:956–961. 10.1210/endo.135.3.8070392 8070392

[B48] Yip SH, Boehm U, Herbison AE, Campbell RE (2015) Conditional viral tract tracing delineates the projections of the distinct kisspeptin neuron populations to GnRH neurons in the mouse. Endocrinology 156:2582–2594. 10.1210/en.2015-1131 25856430

[B49] Yoon H, Enquist LW, Dulac C (2005) Olfactory inputs to hypothalamic neurons controlling reproduction and fertility. Cell 123:669–682. 10.1016/j.cell.2005.08.039 16290037

[B50] Yuste R (2015) From the neuron doctrine to neural networks. Nat Rev Neurosci 16:487–497. 10.1038/nrn3962 26152865

